# A critical guide to the automated quantification of perivascular spaces in magnetic resonance imaging

**DOI:** 10.3389/fnins.2022.1021311

**Published:** 2022-12-14

**Authors:** William Pham, Miranda Lynch, Gershon Spitz, Terence O’Brien, Lucy Vivash, Benjamin Sinclair, Meng Law

**Affiliations:** ^1^Department of Neuroscience, Central Clinical School, Monash University, Melbourne, VIC, Australia; ^2^Monash-Epworth Rehabilitation Research Centre, Turner Institute for Brain and Mental Health, School of Psychological Sciences, Monash University, Melbourne, VIC, Australia; ^3^Department of Neurology, Alfred Hospital, Melbourne, VIC, Australia; ^4^Department of Medicine, The Royal Melbourne Hospital, University of Melbourne, Melbourne, VIC, Australia; ^5^Department of Neurology, The Royal Melbourne Hospital, University of Melbourne, Melbourne, VIC, Australia; ^6^Department of Radiology, Alfred Health Hospital, Melbourne, VIC, Australia; ^7^Department of Electrical and Computer Systems Engineering, Monash University, Melbourne, VIC, Australia

**Keywords:** perivascular space (PVS), MRI, glymphatic system, computer vision, segmentation

## Abstract

The glymphatic system is responsible for waste clearance in the brain. It is comprised of perivascular spaces (PVS) that surround penetrating blood vessels. These spaces are filled with cerebrospinal fluid and interstitial fluid, and can be seen with magnetic resonance imaging. Various algorithms have been developed to automatically label these spaces in MRI. This has enabled volumetric and morphological analyses of PVS in healthy and disease cohorts. However, there remain inconsistencies between PVS measures reported by different methods of automated segmentation. The present review emphasizes that importance of voxel-wise evaluation of model performance, mainly with the Sørensen Dice similarity coefficient. Conventional count correlations for model validation are inadequate if the goal is to assess volumetric or morphological measures of PVS. The downside of voxel-wise evaluation is that it requires manual segmentations that require large amounts of time to produce. One possible solution is to derive these semi-automatically. Additionally, recommendations are made to facilitate rigorous development and validation of automated PVS segmentation models. In the application of automated PVS segmentation tools, publication of image quality metrics, such as the contrast-to-noise ratio, alongside descriptive statistics of PVS volumes and counts will facilitate comparability between studies. Lastly, a head-to-head comparison between two algorithms, applied to two cohorts of astronauts reveals how results can differ substantially between techniques.

## The glymphatic system and perivascular spaces

In the human body, the lymphatic system is the main pathway for waste clearance (Cueni and Detmar, 2008). Generally, lymphatic organs are concentrated in regions of higher energy consumption and production of metabolic waste (Louveau et al., 2018). The lymphatic system, however, does not appear to extend to the nervous system, despite the brain’s vast metabolic demands (Iliff et al., 2013). Instead, the maintenance and waste management of the neuronal environment is governed by the glymphatic system (Hladky and Barrand, 2014; Bakker et al., 2016; Plog and Nedergaard, 2018). Aptly named, the glymphatic system eliminates waste in the brain with a system of glial cells and cerebrospinal fluid (CSF).

**FIGURE 1:**
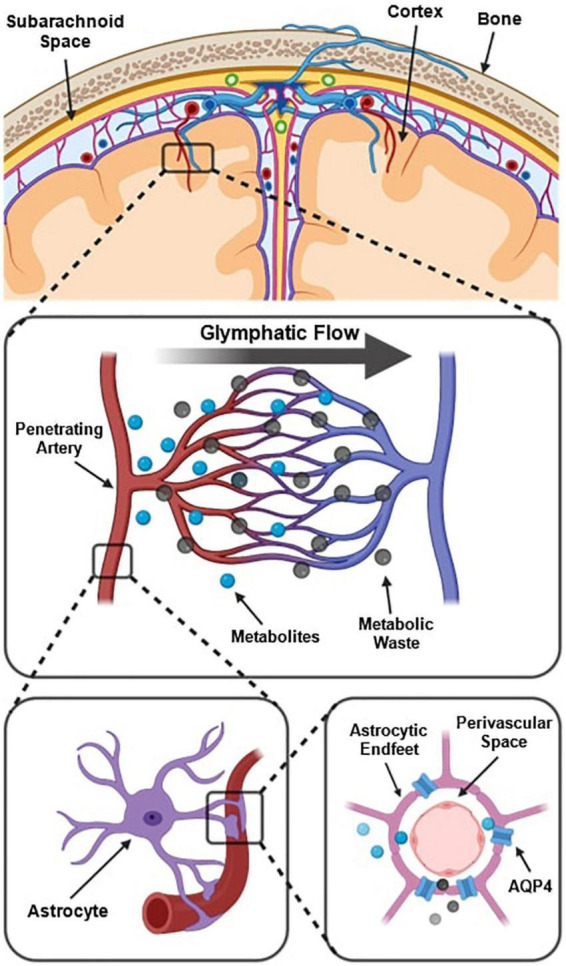
Schematic of the perivascular unit adapted from Troili et al. (2020). **(Top)** Arteries and veins from the subarachnoid space penetrate the parenchyma, perpendicular to the cortical surface. **(Middle)** Metabolites are extruded from arterioles and into the extracellular space, whilst metabolic waste flows towards the perivenular spaces for glymphatic clearance from the neuropil. **(Bottom left)** Astrocytic endfeet enclosing a penetrating arteriole which forms the perivascular space. **(Bottom right)** Aquaporin-4 trans-membrane channels line the astrocytic end-feet and facilitate the exchange of cerebrospinal fluid and interstitial fluid between PVS and extracellular space. Reproduced under the Creative Commons Attribution 4.0 International License (https://creativecommons.org/licenses/by/4.0/).

The prevailing understanding of glymphatic clearance involves the convective fluid flow of CSF through perivascular spaces (PVS), which are formed and lined by glial cells. At the microscopic scale, the glymphatic system comprises perivascular spaces surrounding the cerebral vasculature, also termed perivascular units (Figure 1; Troili et al., 2020). CSF produced in the choroid plexus traverses through the subarachnoid space to irrigate perforating blood vessels. As these vessels penetrate deeper into the cortex, the meningeal layers become continuous with astrocytic endfeet, forming a CSF-filled chamber that encapsulates the cerebral vascular tree (Jessen et al., 2015). The astrocytic end feet densely express aquaporin-4 channels (AQP4) which facilitate the exchange of CSF and interstitial fluid (ISF) between PVS and extracellular spaces (Iliff et al., 2013; Huguenard et al., 2017).

Multiple models have been proposed to explain the fluid flow that occurs in and around perivascular spaces (Hladky and Barrand, 2014, 2022; Bohr et al., 2022). According to one widely held model, CSF flushes out from the periarteriolar spaces, via mechanisms including convective fluid flow and arterial pulsations, into the interstitial space to flush waste metabolites towards perivenular spaces (Figure 1; Troili et al., 2020). At the perivenular spaces, interstitial waste is directed to major areas of CSF clearance and fluid filtration via dural glymphatic drainage pathways such as the venous sinuses (Albayram et al., 2022). This is known as glymphatic flow (Xie et al., 2013; Rasmussen et al., 2018).

### The glymphatic system

Much of our understanding of the glymphatic system stems from animal models. Animal models have provided evidence suggesting that the clearance of metabolic waste in the interstitial space is modulated by sleep/wake states, and that the restorative effects of sleep may largely be due to glymphatic processes. For example, Xie et al. (2013) used a mouse model to demonstrate that the glymphatic system is most active during sleep, compared to awake or anesthetized mice (Xie et al., 2013). Additionally, glymphatic flow appears to be facilitated by shrinkage of the neuronal environment leading to greater interstitial space (Jessen et al., 2015). The importance of AQP4 channels in glymphatic functioning was outlined by Iliff et al. (2012), when AQP4 knock-out mice showed reduced clearance of amyloid-β by 55% and developed memory deficits. Iliff et al. (2012) also showed that this system preferentially mediates the filtration of solutes of smaller molecular size (<3 kDa), having clear implications in neurodegenerative diseases characterized by the harmful accumulation of small neurotoxins such as amyloid-β (Iliff et al., 2012; Huguenard et al., 2017). In disease models, glymphatic functions appear to be attenuated. For example, mouse models of Alzheimer’s disease (AD) exhibited reduced clearance of amyloid-β from the extracellular space (Harrison et al., 2020). Similarly, in a diabetes mellitus mouse model, glymphatic flow was attenuated with delayed clearance of contrast agents from the interstitial space (Jiang et al., 2017; Zhang et al., 2019).

**FIGURE 2:**
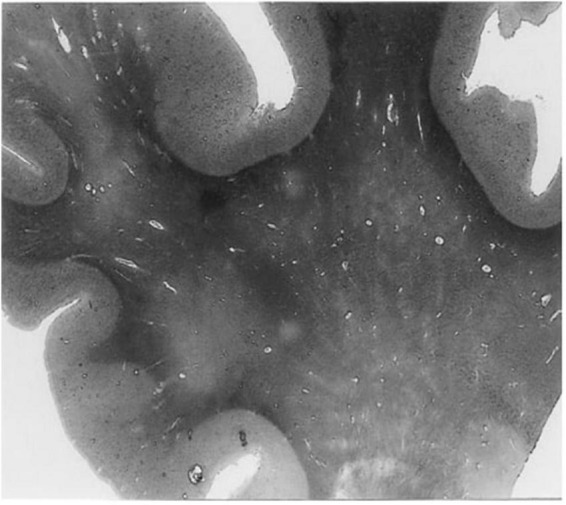
A histological slice of the superior frontal gyrus from an Alzheimer’s disease patient with numerous enlarged perivascular spaces in the white matter (Roher et al., 2003). PVS appear as bright tubular structures and are mainly present in white matter as opposed to gray matter. Adapted from Roher et al. (2003) and reproduced with permission from Springer Nature, conveyed via the Copyright Clearance Center, Inc.

In humans, contrast-enhanced magnetic resonance imaging has been used to show greater clearance of contrast agents shortly after sleep, compared to waking activity, providing further evidence that the glymphatic clearance may be most active during sleep (Lee et al., 2021). Moreover, dysfunctions of the glymphatic system have been associated with many diseases in humans, including cerebral small vessel disease, AD, and multiple sclerosis (Ge et al., 2005; Mogensen et al., 2021; Natário et al., 2021; Benveniste and Nedergaard, 2022). For example, *ex vivo* examination of histological slices from the white matter of an AD patient revealed enlarged perivascular spaces (Figure 2) compared to an age-matched control (Roher et al., 2003). The majority of *in vivo* research in humans, however, uses magnetic resonance imaging to evaluate the extent of perivascular space enlargements.

### Characterization of perivascular spaces

Perivascular spaces were first discovered in the early 1800s and described as état criblé, or diffusely enlarged, for their widespread occurrence in the basal ganglia. They are also known as Virchow-Robin spaces, after Virchow and Robin who hypothesized them to be spaces that are continuous with perineuronal spaces (Woollam and Millen, 1955). Since the arrival of neuroimaging techniques in the late 1980s, enlarged PVS have been observed *in vivo* (Wardlaw et al., 2020).

**FIGURE 3:**
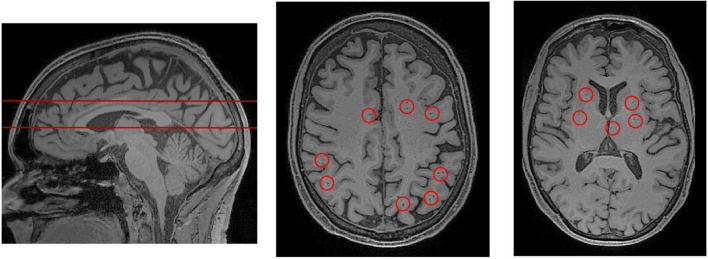
PVS rating scales rely on representative axial slices to assess the severity of PVS enlargement. **(Left)** A sagittal view of the brain, from a T1-weighted MRI scan. The red lines indicate the axial slices that have been selected for visual PVS rating. The upper line was chosen to assess PVS in the centrum semiovale, and the lower line was chosen for the basal ganglia. The centrum semiovale is the mass of white matter above the lateral ventricles. **(Middle)** An axial slice of the centrum semiovale corresponding to the top line. **(Right)** An axial slice of the basal ganglia corresponding to the bottom line. Visible perivascular spaces are outlined by red circles. For an extensive review of rating scales, please refer to Paradise et al. (2020).

**FIGURE 4:**
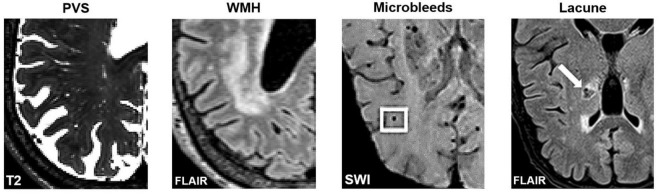
Axial slices of MRI-visible brain lesions, including PVS, white matter hyperintensities, microbleeds, and lacunes. PVS appear as hyperintense and tubular shapes in T2-weighted MRI scans **(Left)**. White matter hyperintensities are prominent on FLAIR images **(Middle-Left)**. Other lesions that can be confused with PVS include microbleeds **(Middle-Right)** and lacunes **(Right)**. In FLAIR scans, lacunes are surrounded by a hyperintense rim, whereas PVS are not. Imaging artifacts such as Gibbs ringing, and motion artifacts can also hinder the automated detection of PVS. Figures of microbleeds and lacunes were adapted from Chesebro et al. (2021) and Li et al. (2019), respectively. The figures are reproduced under the Creative Commons Attribution 4.0 International License (https://creativecommons.org/licenses/by/4.0/). FLAIR, Fluid attenuated inversion recovery; SWI, susceptibility-weighted imaging.

Magnetic resonance imaging (MRI) is the current standard for *in vivo* assessment of PVS in humans. MRI is used to evaluate PVS visibility as a proxy measure of glymphatic dysfunction, and potential occlusion of drainage pathways (Rasmussen et al., 2018). PVSs, particularly when enlarged, are observable in MRI scans (Figures 3, 4; Kwee and Kwee, 2007). Structurally, PVS are long tubular structures that follow cerebral blood vessels (Wardlaw et al., 2013). On MR scans, their appearance depends on the viewing plane and the MRI weighting sequence. On both T1 and T2 weighted images, PVSs are CSF isointense. Thus, they are hypointense or dark on T1 images and hyperintense or light on T2 (Kwee and Kwee, 2007). When viewed along a parallel axis, such as the sagittal or coronal planes, PVS appear as long tubular shapes. On axial slices, PVS appear as small ovoid structures typically less than 3 mm in diameter (Figure 3; Wardlaw et al., 2013). In rare cases, giant tumefactive PVS can exceed 15 mm in diameter (Salzman et al., 2005).

The severity of PVS enlargement is graded by a rater according to established visual rating scales (Figure 3; Patankar et al., 2005; Adams et al., 2013; Potter et al., 2015a; Paradise et al., 2020). Subsequently, these severity scores are associated with features of interest including disease markers and risk factors. Using visual rating scales, the severity of PVS enlargement has been associated with and subsequently proposed as a potential biomarker of various neurodegenerative disorders such as cerebral small vessel disease, AD, neuroinflammation in multiple sclerosis, and cerebral amyloid angiopathies (Hansen et al., 2015; Bakker et al., 2016; Ramirez et al., 2016; Rasmussen et al., 2018; Granberg et al., 2020). Notably, a general weakness of T1 and T2 MRI sequences is that it cannot differentiate between perivenular and periarteriolar spaces (Wardlaw et al., 2020).

Perivascular spaces occur throughout the brain. The most commonly examined regions are the centrum semiovale (CS) and the basal ganglia (BG) (Figure 3). The CS is the mass of white matter (WM) superior to the lateral ventricles, and the BG, which is adjacent to the lateral ventricles and includes the caudate and putamen. Importantly, the anatomy and pathology of PVS are different between regions. In the white matter, blood vessels from the subpial space penetrate the cortical surface and into the parenchyma, are lined by a single leptomeningeal layer: the pia mater (Pollock et al., 1997). In the basal ganglia, penetrating blood vessels are lined by two leptomeningeal layers that connect to the subarachnoid space (Pollock et al., 1997). Thus, the pathophysiology of PVS differ substantially between these two regions (Wardlaw et al., 2020). For a review of the differences between cortical and basal perivascular spaces, please refer to Wardlaw et al. (2020).

Many grading scales have been developed to quantitatively assess the severity of PVS in MRI (Patankar et al., 2005; Adams et al., 2013; Wardlaw et al., 2013; Laveskog et al., 2018; Paradise et al., 2020). The most widely adopted is Wardlaw’s scale which assesses PVS severity in the CS, BG and midbrain (Potter et al., 2015c). According to Wardlaw’s scale, the rater selects a single representative axial slice for each region. If PVSs are observable in the midbrain, it is assigned a score of 1, otherwise it is assigned a score of 0. For the CS and BG, each PVS is counted (Figure 3) and the region is rated according to a 5-point rating scale (0 = no PVS found; 1 = 1–10; 2 = 11–20; 3 = 21–40, 4 = more than 40 present). The 5-point rating scale has high inter-rater and test-retest reliability, and has been used to objectively link PVS and glymphatic dysfunction with markers of disease (Paradise et al., 2020).

### Limitations of visual rating scales

By assigning a simple rating to different counts of PVS, the grading scale is a highly replicable and convenient method of assessing perivascular spaces (Paradise et al., 2020). However, the reduction of PVS counts to a simple severity score restricts deeper analyses of glymphatic dysfunction that may be region-specific or associated with specific morphological changes (Zong et al., 2016; Barisano et al., 2021b). For example, asymmetric distributions of PVS between hemispheres has been related to an increased risk of post-stroke and post-traumatic epilepsy (Duncan et al., 2018; Yu et al., 2022). When there is a large difference in PVS counts between hemispheres, Wardlaw’s scale instructs the rater to use the hemisphere with the higher PVS count (Potter et al., 2015b,c). Moreover, longitudinal analyses of PVS are difficult to perform due to the coarseness of the grading scale, and the use of different scales between publications has led to difficulties comparing results and conducting meta-analyses (Granberg et al., 2020).

**FIGURE 5:**
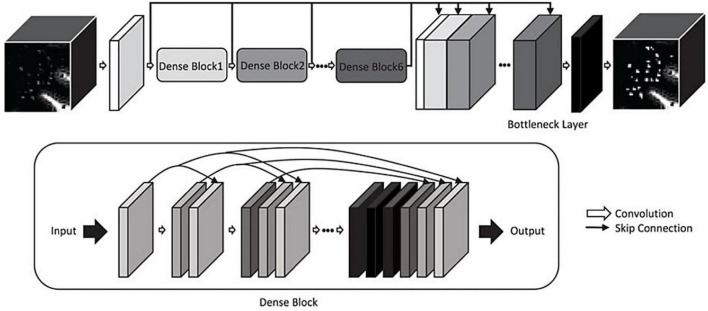
A convolutional neural network for enhancing PVS visibility in T2-weighted MRI images (Jung et al., 2019). © IEEE 2019.

Multiple algorithms have been developed to facilitate or improve the consistency of PVS grading (Descombes et al., 2004; Zong et al., 2016; González-Castro et al., 2017; Dubost et al., 2019a,b; Jung et al., 2019; Sepehrband et al., 2019; Goryawala et al., 2021; Williamson et al., 2022). For example, Jung et al. (2019) applied a convolutional neural network (CNN) to increase the signal-to-noise ratio (SNR) of PVS, thereby enhancing its appearance and improving detection (Figure 5; Jung et al., 2019). Others have automated the quantification of PVS in axial slices to assign severity scores (Dubost et al., 2019a,b). However, these algorithms do not enable the volumetric or morphological examination of perivascular spaces visible in MRI (Valdes Hernandez et al., 2013). Thus, the remaining review will focus on methods for automatically segmenting perivascular spaces.

## Automated segmentation of perivascular spaces

Perivascular spaces are small structures that occur repeatedly throughout the brain. The average volume of a single MRI-visible perivascular space is less than 5 mm^3^, whilst the total PVS volume in a young and healthy individual can average 5,000 mm^3^ (Zong et al., 2016; Barisano et al., 2021b). With a resolution of 1 mm^3^ isotropic (each voxel is 1 mm in length, width, and height), this would require manual labeling of 5,000 voxels to complete PVS segmentation in the average subject. Thus, manual delineation of 3D PVS is laborious and time-consuming. Despite this, the manual segmentation of perivascular spaces is a worthy endeavor for which multiple research groups have dedicated time and resources, in order to develop high quality algorithms (Park et al., 2016; Zong et al., 2016; Zhang et al., 2017; Boespflug et al., 2018; Lian et al., 2018; Schwartz et al., 2019; Smith et al., 2020, Preprint; Sepehrband et al., 2021; Lynch et al., 2022, Preprint). The goal of automatic segmentation algorithms is to label such structures, eliminating the need for manual labeling, thereby expediting detailed analyses of PVS.

Several studies have published automated methods for segmenting perivascular spaces (Table 1; Ramirez et al., 2016; Ballerini et al., 2017; Dubost et al., 2017; Lian et al., 2018). Broadly, these algorithms can be categorized as either (1) classical image processing techniques or (2) machine learning (ML) algorithms. Both have been applied with varying levels of success.

**TABLE 1:** Summary of PVS segmentation methods and their results.

Study	Field strength	Imaging modality	Description	Results
Descombes et al., 2004	1.5T	T1	Reversible jump markov chain monte carlo algorithm.	Count correlation = 0.77 ICC = 0.87
Uchiyama et al., 2008	1.5T	T1, T2	Computer-aided segmentation of PVS after white top-hat transformation.	AUC = 0.945
Ramirez et al., 2015	1.5T	T1, T2, PD	Modified lesion explorer which applies local intensity thresholding to segment lesions (Ramirez et al., 2011).	ρ = 0.38–0.57
Cai et al., 2015	7T	T2	Edge-detection and k-means clustering.	FPs = 10.4 ± 5.0% FNs = 12.7 ± 8.9%
Zong et al., 2016	7T	T1 vs T2	Semi-automated Frangi filtering.	PVS-VF correlations = 0.17–0.74
Wang et al., 2016	1.5T	T2, T2*, T1, FLAIR	Intensity normalization, gamma correction and linear mapping of BG-PVS, followed by manual correction.	Significant association (p < 0.05) between computational counts and visual ratings.
Ballerini et al., 2016, 2017	1.5T	T1, T2	Frangi filtering optimized with ordered logit models.	ρ = 0.67–0.74
Niazi et al., 2018	3T	T2	K-means clustering, edge detection and contrast enhancement.	SEN = 92.9% Specificity = 93.3%
Boespflug et al., 2018; Schwartz et al., 2019	3T	T1, T2 FLAIR, PD	mMAPS: multi-modal autoidentification of perivascular spaces. MAPS: requires only T1 and FLAIR modalities. Applies intensity thresholding and morphological constraints.	Volume correlation r = 0.58 Count correlation r = 0.77–0.87 PPV = 77.5–87.5%
Sepehrband et al., 2019	3T	T1, T2	EPC: Frangi filtering: after NLMF followed by combination of T1 and T2 modalities (EPC).	Inter-class correlation r ≥ 0.83 Scan-rescan correlation r ≥ 0.85
Smith et al., 2020, Preprint	7T	T2	PVSSAS: Perivascular space semi-automatic segmentation tool. Applies Frangi-filtering with a graphical user interface to segment PVS in the white matter.	83% overlap between predicted and manually segmented PVS voxels.
Bernal et al., 2022	–	T2	Comparison between three image filters: Frangi, Jerman, and RORPO.	Median AUC = 94.2–98.7% For images with artifacts: Median AUC = 43.8–71.9%
Park et al., 2016	7T	T1, T2	Random Forest (RF) model with normalized 3D Haar features.	DSC = 64% SEN = 59% PPV = 73%
Dubost et al., 2017	1.5T	PD	GP-Unet for segmentation of BG-PVS.	SEN = 62% FPs per image = 1.5
Zhang et al., 2017	7T	T2	Structured RF with an entropy-based sampling strategy.	DSC = 66% SEN = 65% PPV = 68%
Hou et al., 2017	7T	T2	RF classifier trained with segmentations derived by Haar transformation, block-match filtering and Frangi filtering.	DSC = 67% SEN = 68% PPV = 66%
Lian et al., 2018	7T	T2	M^2^-EDN: Multi-scale, multi-channel encoder-decoder network. A convolutional neural network.	DSC = 77% SEN = 74% PPV = 83%
van Wijnen et al., 2019	1.5T	T2	Geodesic convolutional neural network segmentation from dot annotations.	SEN = 55.3% FPs per image = 5.1
Jokinen et al., 2020	1.5T	T1	uResNet: a U-shaped convolutional neural network with residual elements (Guerrero et al., 2018).	Volume correlation = 0.91
Boutinaud et al., 2021	3T	T1	3D U-net with an autoencoder.	DSC in the WM = 51% DSC in the BG = 66%
Spijkerman et al., 2022	7T	T1, T2-TSE	K-nearest neighbor classifier trained with sato filtered segmentations (white matter only).	DSC = 61% ICC = 0.74 Inter-rater DSC = 49%
Sudre et al., 2022, Preprint	3T	T1, T2, FLAIR	VALDO: a competition between PVS segmentation algorithms which included three convolutional neural networks and a random forest (RF). The RF outperformed the other three models.	Median (IQR) DSC^†^ Inter-rater = 19.6 (13.5–23.8) % RF performance = 38.9 (28.9, 49.4) %
Rashid et al., 2022 Preprint	3T	T1, T2, SWI, FLAIR	Multi-channel 2D U-nets trained with data augmented axial slices.	SEN = 82% PPV = 84% ICC (volume) = 0.67

Listed first are the methods that utlize classical image filtering technique. In the second section are methods that have applied machine learning techniques. Reported here are the mean or range of performance metrics published by the original authors, unless otherwise specified. ^†^Dice similarity coefficient derived with the six-neighborhood connected components rule. The Dice score is also known as the F1 score. FLAIR, fluid attenuated inversion recovery sequence; PD, proton density sequence; SWI, susceptibility weighted imaging; TSE, turbo-spin-echo; ICC, intra-class correlation coefficient; AUC, area under the receiver operating characteristic curve; FP, false positive; FN, false negative; VF, volume fraction; DSC, Sørensen-Dice similarity coefficient; SEN, sensitivity; PPV, positive predictive value; VALDO, vascular lesions detection and segmentation challenge; RF, random forest; NLMF, non-local means filtering; PVSSAS, perivascular space semi-automatic segmentation tool; RORPO, ranking orientation responses of path openings; EPC, enhanced perivascular contrast.

**FIGURE 6:**
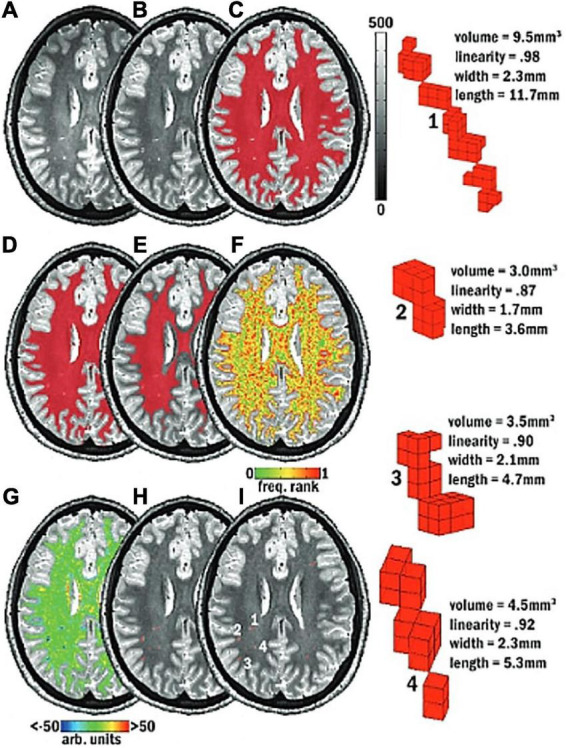
Schematic of the mMAPS (multi-modal auto-identification of perivascular spaces) pipeline for the automated segmentation of PVS adapted from Piantino et al. (2020). It relies on the sequential application of image filters, intensity thresholds, and morphological constraints to delineate PVS voxels. **(A)** A T2-weighted MRI image is acquired. **(B)** Voxel intensities are normalized. **(C)** White matter (WM) mask (red) applied. **(D)** Holes in the WM mask are filled. **(E)** Edges of the WM mask are eroded. **(F)** Frequency map of the voxel intensities. **(G)** Map of the local intensity differences. **(H)** Seed clusters resulting from the previous steps are extracted and filtered with morphological constraints. **(I)** The final segmentation map. Reproduced with permission of the American Society of Neuroradiology, from Piantino et al. (2020), conveyed through the Copyright Clearance Center, Inc.

In the classical approaches, a computerized pipeline with explicit parameters is set-up and optimized to search for PVS (Ballerini et al., 2016, 2017). For example, seed clusters selected based on intensity thresholds within the white matter can be filtered based on their size, linearity, length, and width (Figure 6; Boespflug et al., 2018). One disadvantage of these methods is that they often require further optimization for different datasets depending on the imaging protocols (Ballerini et al., 2016).

In comparison, ML-based algorithms undergo training with example data to perform a certain task (Bengio, 2013). In PVS segmentation, the algorithm is trained with manually labeled images, over many iterations or epochs, to learn the features associated with PVS. After training, ML algorithms can be used to label PVS structures on new and unseen data. Although the initial stages are computationally expensive, the results are generally worthwhile as ML algorithms can learn complex features that cannot be defined via classical techniques (Bengio et al., 2012).

### Evaluation of segmentation performance

In the evaluation of image segmentation algorithms, the true positives, false positives, and false negatives are tallied to compute three main metrics: the Sørensen–Dice coefficient or F1 score, the sensitivity or detection rate, and the positive predictive value (PPV) (Equation 1) (Dice, 1945; Lian et al., 2018; Jung et al., 2019; Siddique et al., 2021).


$$D\,S\,C=\frac{2\,T\,P}{2\,T\,P+F\,P+F\,N};S\,E\,N=\frac{T\,P}{T\,P+F\,N};P\,P\,V=\frac{T\,P}{T\,P+F\,P}$$

Equation 1. DSC, Sørensen–Dice similarity coefficient; SEN, sensitivity; PPV, positive predictive value; TP, true positives; TN, true negatives; FP, false positives; FN, false negatives.

The Dice score is a measure of overall segmentation performance (Dice, 1945). A higher Dice score means better overall performance. The sensitivity is the percentage of all PVS voxels that are detected by the algorithm. Whereas the PPV is the percentage of predicted voxels that were correctly labeled as PVS. These metrics assess different aspects of the algorithm’s ability to delineate perivascular spaces. Together, they are useful for gauging the tendencies of an algorithm’s segmentation ability. For example, higher sensitivity than PPV indicates the algorithm prioritizes detection over accuracy. Whereas higher PPV vs. sensitivity indicates the algorithm prefers accuracy or correctness over detection.

However, the Dice coefficient is not infallible. A common problem in medical image segmentation tasks, and especially PVS segmentation is that of noisy labels (Karimi et al., 2019). Due to a number of reasons such as poor image quality, rater fatigue or time constraints, the ground truth can encompass a number of false positives and false negatives that affects that Dice coefficient (Wardlaw et al., 2013; Moses et al., 2022). Thus, the sources of error that affects the Dice score are:

(1) Error in the ground truth (human labels).
(2) Error in the algorithm prediction.

The Dice score aims to measure the latter, but if the ground truth is not reliable, then its ability to do so is impaired, and resultant Dice scores are distorted. In the ideal scenario, the ground truth is perfect and therefore any decrement in the Dice score is purely due to algorithm error. However, to attain this perfect ground truth would require high inter and intra-rater agreement, and robust standards for PVS delineation. To a great extent this has been achieved with 2D rating scales, e.g., STRIVE and UNIVERSE, demonstrating high-concordance across research groups (Wardlaw et al., 2013; Adams et al., 2015). Currently, there is no guideline for the voxel-wise segmentation of perivascular spaces in either T1 or T2-weighted MRI data.

Variations of the Dice metric have been employed, such as the Dice score with 6-neighborhood connected components rule, wherein predicted PVS voxels are deemed true-positives if they are adjacent to a real PVS voxel (Sudre et al., 2022, Preprint). These do not address the inherent issue of noisy labels in PVS segmentation, rather they merely inflate the reported scores. Such modifications make it difficult for comparisons to be made to similar studies that have employed the traditional Dice metric (Equation 1). It also obfuscates the fact that PVS segmentation is a more difficult task compared to other medical imaging problems, where inter-rater Dice scores of 70%+ are commonplace, as opposed to PVS segmentation where inter-rater Dice scores are usually below 50% (Sudre et al., 2022, Preprint; Liu et al., 2022; Spijkerman et al., 2022). This is likely due to the nature of the task, as PVS are small, numerous and occur repeatedly throughout the brain, whilst other lesion detection tasks such as for tumors or stroke lesions require delineation of a single, large and prominent object. Thus, the Dice score is not a perfect metric, but it is the most rigorous one for the evaluation of algorithm performance in PVS segmentation.

To our knowledge, no study has directly addressed this problem of noisy labels in PVS segmentation. One review that has examined solutions to address noisy labels in medical images, suggest the use of semi-automatic annotations, wherein models are trained on incomplete data, then used to aid the human in the detection of undetected case (Karimi et al., 2019).

Several studies have employed semi-automated methods, e.g., with the Frangi filter, to efficiently generate PVS maps (Park et al., 2016; Sepehrband et al., 2021; Langan et al., 2022; Ranti et al., 2022). Subsequently, these masks can be compared to the raw predictions to derive Dice similarity coefficients, and thus measure model performance. The downside is that segmentation performance is likely to be overestimated, compared to Dice scores derived with independently generated manual segmentations.

Whilst the evaluation metrics may be biased, the practical benefit is that considerable amounts of time and resources are saved, since correction of automated segmentations may be less time-consuming than purely manual segmentations. Whether this advantage outweighs the disadvantage of a biased model evaluation should be up to the individual researcher. Interestingly, if a machine learning model is trained on a small subset of purely manual PVS labels, and subsequently used to generate a large sample of semi-automated segmentations, then the bias might be diminished, compared to semi-automated Frangi labels, since the model was originally trained on purely human labels.

**FIGURE 7:**
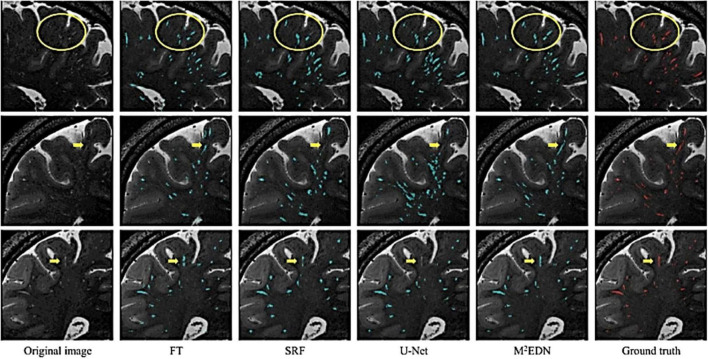
Visual assessment of predicted PVS segmentations from different algorithms to the ground truth, adapted from Lian et al. (2018). Visual comparisons are used to compare algorithms to each other and the ground truth. Red voxels are PVS that have been manually labeled by a human rater. Cyan voxels were predicted to be PVS by the respective automated method. Yellow arrows and circles indicate low contrast perivascular spaces that can be used to differentiate segmentation ability of different methods. FT, Frangi filtering; SRF, structured random forest; M^2^EDN, multi-channel, multi-scale encoder-decoder network. Reprinted from Lian et al. (2018), with permission from Elsevier, conveyed through the Copyright Clearance Center, Inc.

Furthermore, visual comparisons between model predictions and the ground truth are often conducted (Figure 7). Certain weaknesses of the algorithm can then be uncovered, and the algorithm adjusted retrospectively. Visual inspection is important, since PVS can be easily confounded with other lesions or imaging artifacts including lacunes, white matter hyperintensities (WMH) and microbleeds (Figure 4; Wardlaw et al., 2013; Lian et al., 2018).

Another common method of validating segmentation performance is to compare quantitative measures of PVS produced from segmentations, such as total PVS volumes or counts, with conventional PVS ratings (Boespflug et al., 2018; Schwartz et al., 2019). A high correlation coefficient is expected and ensures newer algorithms are commensurate with established methods. Importantly, the aforementioned methods of assessing algorithm performance are not infallible and can be misleading when incorrectly applied. These will be further discussed in more detail alongside recommendations to avoid such problems.

### Existing approaches

An example of a traditional approach to segmentation is the multimodal auto-identification of perivascular spaces (mMAPs) algorithm by Boespflug et al. (2018). This method determines the likelihood of a voxel being a PVS, based on its intensity on co-registered T1, T2, FLAIR (Fluid attenuated inversion recovery) and Proton density (PD) weighted images. Voxels exceeding a certain probability are grouped into clusters, which are then deemed to be PVS if they are sufficiently linear in shape. Their application demonstrated strong correlations to visual rating scores by experts (*r* > 0.65) (Boespflug et al., 2018). Importantly, mMAPS required four imaging modalities: T1, FLAIR, Proton Density, and T2 sequences.

Schwartz et al. (2019) adapted the mMAPS algorithm to segment perivascular spaces using only two imaging modalities (T1-weighted and FLAIR images) from the Alzheimer’s Disease Neuroimaging Initiative (ADNI) dataset (Jack et al., 2008; Boespflug et al., 2018; Schwartz et al., 2019). Both the correlation coefficient, comparing PVS volumes to visual PVS ratings (*r* > 0.7), and PPVs (77.5–87.5%) were high. Worth noting is that neither Dice coefficients nor sensitivity scores were published. PPV alone is an incomplete assessment of the algorithm. For example, if an algorithm correctly labeled 100 voxels in an image with 1000 PVS voxels, its PPV would be 100%, neglecting the remaining 900 voxels it missed resulting in a Dice coefficient of only 18%. Nevertheless, the application of mMAPS to commonly acquired T1-weighted images marks an important step towards automated PVS segmentation in clinical settings.

Another common approach utilizes image filters to highlight perivascular spaces. In this context, filters are operations that calculate the resemblance of voxels and its neighbors, to features of interest such as tubes or edges. For example, the Frangi filter calculates the “vesselness” of voxels after taking into account surrounding values in order to highlight vessel-like structures (Frangi et al., 1998; Ballerini et al., 2016).

**FIGURE 8:**
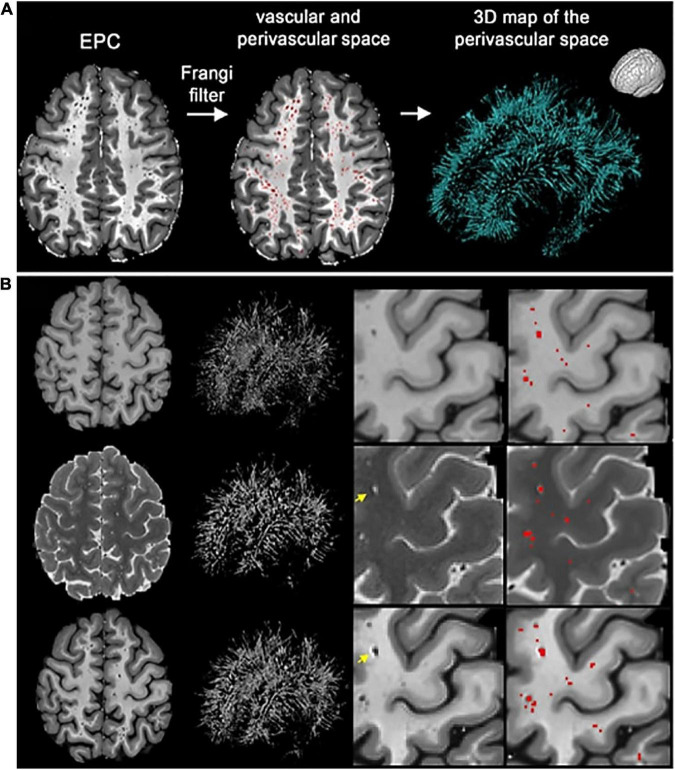
The enhanced perivascular contrast (EPC) pipeline proposed by Sepehrband et al. (2019). In EPC, a T1-weighted image is combined with a co-registered T2-weighted image to improve the visibility of PVS. **(A)** Subsequently, non-local means filtering is applied and PVS are automatically segmented by a Frangi filter. **(B)** Comparisons between Frangi filtered PVS from a lone T1 image (top), a lone T2 image (middle), and combined modalities in EPC (bottom). Segmented voxels are labeled in red. Reproduced under the Creative Commons Attribution 4.0 International License (https://creativecommons.org/licenses/by/4.0/).

One notable approach combined both T1 and T2 scans from a single subject to produce an image with enhanced perivascular contrast or EPC (Figure 8; Sepehrband et al., 2019). By dividing the T1 image by its co-registered T2-weighted counterpart, the contrast between tissue types is enhanced, and therefore PVS clusters more discernible from the white matter. Subsequently, optimized Frangi filtering was applied to automatically label PVS voxels (Frangi et al., 1998; Ballerini et al., 2016). With EPC, the PVS-to-white matter contrast was substantially greater than in either the T1 or T2 image alone, and the number of manually detected PVS was significantly increased (Sepehrband et al., 2019). However, compared to T1 or T2 alone, EPC did not significantly improve count correlations with expert evaluations (Sepehrband et al., 2019).

Typically, Dice scores are used to compare the prediction of an algorithm to a ground truth that was generated independently, usually by manual human segmentation. However, in this study, the strong Dice scores reported (74–95%) were comparing the predicted PVS maps before and after manual correction (Sepehrband et al., 2021). Therefore, these scores are biased and should be expected to perform slightly worse when compared against independently produced segmentations.

Importantly, Sepehrband et al.’s (2021) reported Dice scores highlight how the metrics can be misleading. Dice scores of algorithm predictions before and after manual correction resulted in an average of 95% (Sepehrband et al., 2021). However, when the segmentations were further corrected aided by FLAIR images, this resulted in an average Dice score of 74%. FLAIR images are often used to differentiate PVS structures from confounds such as WMH and lacunes, thus are able to identify false positives (Wardlaw et al., 2013). Clearly, the quality of the segmentations has improved after FLAIR-accompanied corrections, yet the Dice metric has declined by more than 20% (Sepehrband et al., 2021). The implication here is that, without an accompanying FLAIR image the PVS measurements would have been inflated by false positives, via inclusion of WMHs mistaken as PVS. It also suggests Frangi filtering may not be suitable for investigating PVS in disease cohorts without the additional FLAIR modality (Frangi et al., 1998; Ballerini et al., 2016). Notably, the correction for FLAIR WMHs may have removed PVS that reside within the WMHs.

### Machine learning approaches

Machine learning algorithms differ from classical methods in that they automatically learn the features of their target object, such as its shape, intensity, and location. The main ML models that have been applied to PVS segmentation are random forests and CNN.

The first instance of machine learning for automated segmentation of PVS, was done by Park et al. (2016) using a random forest model. With the help of a Frangi filter, 17 MRI images were manually segmented and used to train random forest models. Compared to models trained with intensity thresholded images or vessel-ness filtered images, the random forest performed best when trained on normalized Haar filters, enabling it to learn discriminative PVS features (Park et al., 2016). Dice scores for the optimal model averaged 64%. Sensitivity was lower at 59%, and PPV was 73%. Importantly, high-resolution MRI images, 7 Tesla (T), with both T1 and T2 modalities were used. The labels used to train the random forests were initiated using a Frangi filter, then refined manually, i.e., semi-automatically. As such the resultant random forests would likely be biased to detect voxels more strongly detected by the Frangi filter.

Similarly, Zhang et al. (2017) used a structured random forest to delineate PVS in 7T images. Here, three different filters based on vascularity were used to differentiate PVS from background voxels. This was supplemented with an entropy-based sampling strategy to select regions of interest from the image. Similar to Park et al. (2016), an average 66% Dice score, 65% sensitivity and 68% PPV was achieved (Zhang et al., 2017).

**FIGURE 9:**
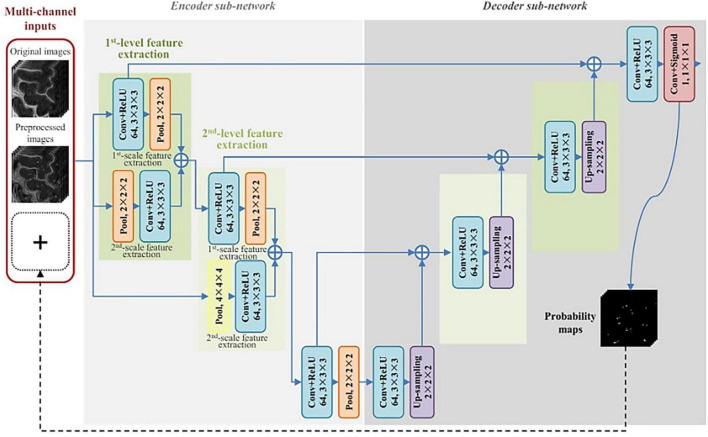
The M^2^EDN neural network architecture proposed by Lian et al. (2018). M^2^EDN stands for multi-scale, multi-dimensional encoder-decoder network. The model features a Frangi filter segmentation as a second input channel. Conv, convolution; ReLU, rectified linear unit; Pool, max pooling. Reprinted from Lian et al. (2018), with permission from Elsevier, conveyed through the Copyright Clearance Center, Inc.

The first instance of automated PVS segmentation using a fully CNN was by Lian et al. (2018). Lian et al. (2018) applied a multi-scale and multi-channel CNN architecture for this task, named the M^2^EDN (Figure 9). The multi-scale feature enables the model to incorporate both small and large contextual details to improve PVS detection. Frangi filtering was also used as a secondary input channel, providing more information to the model, and lastly initial predictions from the network were used as a third channel of information to refine predictions in the With 7T, T2-weighted data achieved an average Dice score of 77% was achieved (sensitivity = 74%, PPV = 83%), superseding the previous machine learning approaches with similar data parameters (Lian et al., 2018).

To extend this work, it is necessary to explore methods of automated segmentation with lower quality datasets that are more accessible than 7T images. Boutinaud et al. (2021) employed another CNN called the u-net with an autoencoder to segment 3T, T1 images (Boutinaud et al., 2021). Typically, model parameters are initialized randomly. However, an autoencoder enables meaningful initialization of model parameters to optimize the performance of the final model (Kingma and Welling, 2013). In this case, it is unclear whether the inclusion of an autoencoder improved performance. Trained on 40 manually labeled images, the model achieved a voxel-wise Dice score of 51% in the white matter and 66% in the basal ganglia. Notably, for PVS clusters larger than 10 mm^3^, Dice scores above 90% could be reliably achieved (Boutinaud et al., 2021).

The noticeable decrement in performance compared to previous ML approaches can be attributed to the lower image quality and resolution of the data (7T vs. 3T) (Lian et al., 2018). However, Wang et al. (2021) recently published a study applying Lian et al.’s (2018) CNN to 3T, T2-weighted data with a Dice score of 70%. A similar performance level was achieved when the same CNN was applied to T1-weighted images, suggesting that the multi-channel and multi-scale architecture is superior to a regular u-net with an autoencoder (Kingma and Welling, 2013; Huang et al., 2021). For an in-depth discussion of each of the techniques, please refer to previous work (Barisano et al., 2022a; Moses et al., 2022).

### Limitations of automated segmentations

With the automated delineation of perivascular spaces, new ways of understanding the glymphatic system are possible. However, these studies are based on algorithms with certain disadvantages. The conventional methods relying on image processing techniques require further parameter optimization for different datasets (Ballerini et al., 2016; Smith et al., 2020, Preprint; Boutinaud et al., 2021; Bernal et al., 2022). Currently, only one fully automatic segmentation pipeline is freely available, making it difficult to replicate previous methods (Boutinaud et al., 2021). Most machine learning approaches were trained or tested with data from high field (7T) MRI scanners that are not commonly used clinically (Park et al., 2016; Zhang et al., 2017; Lian et al., 2018). Several algorithms require multiple imaging modalities (Boespflug et al., 2018; Sepehrband et al., 2021). However, the primary MRI sequences acquired are T1 or T2-weighted images, with FLAIR sequences less commonly acquired (Schwartz et al., 2019).

Currently, there is no gold standard for automated 3D PVS segmentation in either T1 or T2 data leading to highly heterogenous methodologies and results across research groups. The issue is further compounded by the different scanner protocols and processing methods being utilized. Factors affecting the SNR and contrast-to-noise (CNR) ratios of PVS, including field strength, signal weighting, or resolution will attenuate PVS detection (Zong et al., 2016). Therefore, PVS detection techniques applied to either T1, T2, or both, and for different image qualities, are yet to be adopted as the gold standard.

The ideal PVS auto-segmentation tool should meet the following criteria:

(1) Open source and freely available, alongside optimized parameters or trained weights for datasets of different image qualities. Publication of algorithms in code repositories such as Github enable external reproducibility.
(2) High performance in regards to voxel-wise evaluations including Dice coefficients, sensitivity and PPV metrics based on ground truth manual segmentations.
(3) Able to segment PVS throughout the brain, including the gray and white matter, basal ganglia, hippocampus, and brainstem.
(4) Robust to noise and able to differentiate PVS from structurally similar objects such WMH, lacunes, microbleeds, and imaging artifacts (Figure 4).

### Recommendations for model development

To objectively adhere to the criteria, we make the following recommendations, which relate to establishing the validity, robustness and reproducibility of the model.

(1) Validate segmentation performance on multiple open access datasets.
(2) Benchmark against previously published and optimized algorithms.
(3) Evaluate against high quality manual segmentations.
(4) Publish PVS voxel counts and volumes, image quality metrics including CNR, field strength, and voxel size as descriptive variables.
(5) Report all relevant segmentation metrics: Dice scores, sensitivity and PPV.
(6) Evaluate algorithm robustness on a noisy dataset, including images with MRI artifacts, and PVS mimics, e.g., lacunes, microbleeds, and WMHs (Wardlaw et al., 2013).
(7) Publish visual examples of algorithm predictions with 3D renders and 2D slices, displaying PVS both cross-sectionally and lengthwise. Examples should be either the default contrast that was input into the algorithm or contrast adjusted to improve the visibility of PVS.

Points 1–2 relate to establishing the reproducibility and benchmarking of the algorithm. Validation of algorithm should be completed on multiple open access datasets of inhomogeneous image qualities. Segmentation of PVS can be conducted in tools such as ITKSnap, Freeview, or Osiris (Yushkevich et al., 2006; Fischl, 2012; Othman et al., 2016). Publication of manual segmentations and predictions on open access datasets would contribute significantly towards progress in PVS research. Moreover, publishing source codes and pipelines would enable fair comparisons and benchmarking of PVS detection methods, allowing for direct comparisons between approaches. In this regard, ML approaches with trained weights should be compared to fully optimized versions of traditional methods, e.g., the Frangi filter, for a fair comparison.

Points 3–5 relate to validating the performance of the algorithm. Currently, as there is no consensus for automated methods of PVS segmentation. The gold standard of manual segmentation should be used as a benchmark for judging algorithm performance. Semi-automated methods of model validation may result in inflated and biased Dice scores (Sepehrband et al., 2021; Langan et al., 2022; Ranti et al., 2022). Another reason for inflated Dice scores may be the under-labeling of PVS, which lowers number of PVS that the algorithm is required to detect. This can be observed by lower-than-expected average counts of PVS voxels and volumes. In the same way a study publishes the sample size, and the percentage of subjects that developed a disease, publishing counts of PVS voxels in the ground truth would make the resulting Dice scores more meaningful. An algorithm that performs well on images with a small number of PVS may not perform as well for MRI scans with greater volumes of PVS.

**FIGURE 10:**
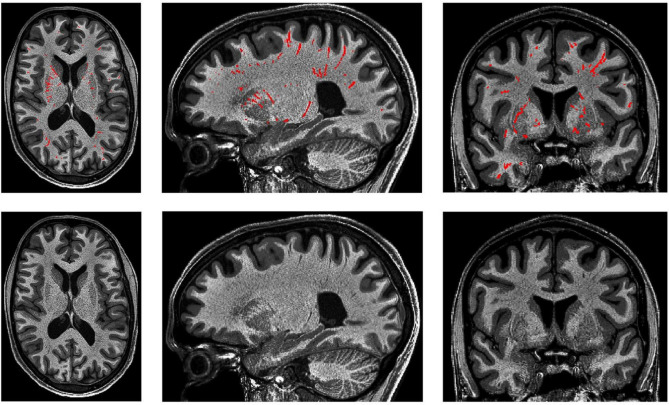
3D PVS segmentation from a T1-weighted MRI scan. PVS voxels are labeled in red (top row), and the corresponding unlabeled slices are in the bottom row. Basal ganglia PVS are visible in the axial (left), sagittal (middle), and coronal (right) slices.

Noisy labels are inherent in neuroimaging segmentations. Many factors can cause PVS voxels to go undetected by manual raters, including experience, time constraints, image quality and contrast, and the inclusion/exclusion criteria. Therefore, an experienced neuroradiologist should validate the integrity of ground truth segmentations. A detailed guide for detecting PVS in T2 MRI images has been published by Potter et al. (2015c). As this guide primarily focuses on T2-weighted axial slices for the purpose of assigning PVS enlargement scores according to a rating scale, we make further recommendations to aid segmenters in producing high-quality 3D PVS segmentations:

(1) All voxels within a cluster should be labeled such that no hypointense or hyperintense voxels connected to that cluster remain visible, in T1 or T2-weighted images, respectively.
(2) Where there are cerebral blood vessels there are PVS, including the white matter, gray matter, basal ganglia, hippocampus, and brainstem (Figure 10).

(3) In the white matter, PVS are generally directed towards the ventricles (Figure 10).
(4) In the basal ganglia, PVS following the lenticulostriate arteries are most obvious in the sagittal views, appearing to travel superiorly with an anterior curvature (Figure 10).
(5) To distinguish PVS from WMHs, where available, FLAIR images should be used (Wardlaw et al., 2013). WMHs are visible on all three sequences: T1 (as hypointense), T2, and FLAIR. On FLAIR sequences, the PVS are not visible but WMHs are.

Given their numerosity and at times inconspicuous appearance of PVS in MRI, inexperienced raters may find it difficult to detect and label PVS clusters in their entirety (Wardlaw et al., 2013). PVS clusters should be labeled when there is sufficient evidence based on features such as voxel intensity, cluster size (e.g., >4 voxels), directionality, and brain region. Additionally, segmentation software such as ITKSnap allow for manual contrast adjustments (Yushkevich et al., 2006). Contrast adjustment can be very useful, as varying contrast or brightness levels may be required for different regions of the image, e.g., due to bias field inhomogeneities. To improve PVS visibility in one region of the image may require brightening, whereas other regions may require darkening.

The visibility of PVS is strongly associated with image quality. The field strength of the MRI scanner has been shown to significantly affect PVS detection (Barisano et al., 2021a). However, this does not take into account preprocessing methods where images are enhanced to increase PVS visibility. For example, by combining 3T T1 and T2 images in the EPC pipeline, it is likely that the CNR for PVS are greater than 7T data without preprocessing (Sepehrband et al., 2019). Ultimately, CNR is the most direct and comparable metric of PVS visibility for a given dataset, considering variables including scanner field strength, and preprocessing methods. Publication of SNR and CNR values are useful for determining whether appropriate comparisons between studies with different datasets can be made, since models developed on data of lower SNR may not be applicable to higher SNR datasets. This will be further discussed in section “Recommendations for future research and clinical applications.”


$$S\,N\,R=\frac{M\,E\,A\,N_{G\,M}}{S\,D_{A\,I\,R}};C\,N\,R=\frac{M\,E\,A\,N_{W\,M}-M\,E\,A\,N_{G\,M}}{S\,D_{A\,I\,R}}$$

Equations 2. SNR, signal-to-noise ratio; CNR, contrast-to-noise ratio; MEAN_*GM*_, mean gray matter intensity; SD_*AIR*_, standard deviation of the intensity or empty voxels outside the head; MEAN_*WM*_, mean of the white matter intensity. These metrics should be calculated with respect to PVS voxels and within the bounds of the 3D brain extracted volume. These equations apply for T1-weighted images. For T2-weighted images, the MEAN_*WM*_ and MEAN_*PVS*_ are interchanged.

Alternative methods of assessing segmentation performance include PVS count correlations or interclass concordance correlations. These are convenient assessments of reliability and accuracy of PVS cluster quantification, but not voxel-wise segmentation performance, and therefore voxel-based volumetric assessments of PVS. One critique of the Frangi filter is that its performance deteriorates as the size of a PVS cluster increases (Bernal et al., 2022). The implication for PVS research is that PVS counts, rather than volumes, are more likely to demonstrate statistical differences. Thus, count correlations for validation of segmentation performance do not convincingly demonstrate high PVS detection at a voxel-level, and Dice metrics should be preferred.

Findings from related fields of neuroimaging and lesion detection can offer useful insights applicable to PVS segmentation. In MRI lesion segmentation of patients with multiple sclerosis, inter-rater dice scores (between two human raters) around 60% are typical (Egger et al., 2017). For these tasks, there are a small number of large lesions to be detected in a single brain scan. In comparison, PVS occur repeatedly and throughout the brain, numbering in the hundreds. Given that PVS are more numerous and diffuse, one would expect that inter-rater dice scores for the task of PVS segmentation to be much lower. Two publications have assessed inter-rater agreement in PVS segmentation, with median or average inter-rater Dice scores of 26.7 and 49% (Spijkerman et al., 2022) implying that segmentation algorithms exceeding this 50% Dice threshold has superseded human performance (Spijkerman et al., 2022). Necessarily, visual inspections should be conducted to verify that the model outperforms the human labels. Broadly, the algorithm should be critiqued based on its ability to consistently detect all PVS clusters labeled by the human, and whether it can reliably label all PVS voxels belonging to each cluster. In the ideal scenario, where the human segmentation is free of error and imperfection, a Dice score of 100% would indicate a perfect prediction. In practice, the segmentation of medical images often includes noisy labels, i.e., false positives and false negatives, thus such Dice scores are not realistic.

If a model has genuinely outperformed manual segmentations, then presumably it has labeled not only all PVS detected by the rater, but also PVS that evaded human detection. In this case, a high Dice score should be observed alongside a very high sensitivity score and a lower PPV, as the model is presumably detecting more PVS clusters than were labeled manually. This may indeed be the case for Sepehrband et al.’s (2021) approach with non-local means filtering and the Frangi filter (Dice = 74%, Sensitivity = 98%, PPV = 61%) (Figure 8). Thus, all three metrics serve complementary roles to assess model predictions. Furthermore, recording Dice scores for different regions ensures the model performs adequately throughout the brain white matter, basal ganglia, midbrain, and hippocampus. Performance in one region might not be indicative of performance in another region as the appearance and anatomy of PVS in the white matter differ substantially from PVS in the BG (Figure 10). Not only are these regions different structurally, but the morphology of PVS may differ between regions. For example, PVS inferior to the putamen tend to be larger in width and volume, then those that are above the level of the putamen (Pullicino et al., 1995; Wardlaw et al., 2013; Bouvy et al., 2014).

Moreover, testing the robustness of the algorithm in noisy data with image artifacts and PVS confounds will be useful. This is especially important when the algorithm is to be applied in disease cohorts, where such lesions and PVS mimics are commonly observed. If algorithms are to be useful in characterizing PVS in disease, they need to be validated in similar data. Almost all algorithms published were developed based on “clean” images without PVS mimics that commonly occur in patients of neurodegenerative diseases. To our knowledge, only Sepehrband et al. (2021) has evaluated the effect of WMH presence on the Frangi filter. Co-registered FLAIR scans were used to manually correct PVS masks generated by the Frangi filter, and found a substantial decrement of 21% in the Dice metric (Sepehrband et al., 2021). Thus, if findings in pathological cohorts derived from these algorithms are to be trusted, they need to be validated against noisy and pathological data.

### Key biological findings from perivascular spaces segmentations

Over the course of the lifespan changes in gray matter, white matter, and ventricular volumes are numerous and have been well documented (Bethlehem et al., 2022). The growth trajectory of perivascular spaces, visible in MRI, has not been as comprehensively characterized, but insights can be gleaned from multiple studies (Table 2).

**TABLE 2:** Summary of the PVS research that has resulted from 3D segmentations.

Study	Cohort description	MRI sequences, segmentation method	Findings	PVS measures, mean (SD)
Ramirez et al., 2015	AD patients (*n* = 203, mean age = 72.7 years), controls (*n* = 94, mean age = 69.5 years).	1.5T, T1, T2, Proton density. Modified lesion explorer.	WM-PVS volumes were significantly greater in AD patients than controls. Men had larger WM-PVS volumes than women but not BG-PVS volumes.	WM-PVS volume AD: 31.7 (48.4) mm^3^ Controls: 21.7 (31.8) mm^3^
Berezuk et al., 2015	Healthy subjects (*n* = 26, mean age = 20 years).	3T, T1, T2. Modified lesion explorer.	Sleep efficiency was negatively correlated with PVS volume and BG-PVS volume. BG-PVS was negatively correlated with duration of N3 sleep phase.	WM-PVS volume: 10.8(16) mm^3^ BG-PVS volume: 56.4 (63.7) mm^3^
Cai et al., 2015	AD patients (*n* = 5, mean age = 78.2 years); controls (*n* = 3, mean age = 69.3 years).	7T, T2. Edge-detection, k-means clustering and other image processing techniques.	PVS VF was significantly larger in AD patients compared to controls.	WM-PVS VF AD: 8.0 (2.1) % Controls: 4.9 (1.3) %
Zong et al., 2016	Healthy subjects (*n* = 7, age range = 21–37 years).	7T, T1, T2. Semi-automated Frangi filtering.	Mean length of PVS clusters differed significantly between regions. PVS diameter was larger in subcortical nuclei (including the basal ganglia and thalamus) than WM regions. PVS VF also differed between WM regions.	N/A
Wang et al., 2016	Mild stroke patients (*n* = 100, mean age = 69 years, range = 37–92 years).	1.5T, T1, T2, T2*, FLAIR. Gamma correction, intensity thresholding and manual correction.	BG-PVS volumes and cluster counts were associated with WMH volumes and Fazekas ratings. BG-PVS volumes and cluster counts were negatively correlated with brain volume (expressed as a % of ICV).	BG-PVS, median (range) Counts: 10 (0–30) Volumes: 0.093 (0–0.4) mL
Niazi et al., 2018	MCI (*n* = 14, mean age = 71.9); controls (*n* = 15, mean age = 66.3 years).	3T, T2. K-means clustering combined with image processing.	WM-PVS VF was significantly larger in MCI patients compared to controls.	WM-PVS VF Controls: 2.82 (0.4) % MCI: 4.17 (0.57) %
Piantino et al., 2020	Adolescents (*n* = 118, age range = 12–21 years).	3T, T1, T2. mMAPS.	PVS were bilaterally symmetric and were more common in the frontal and parietal lobes. Males had more PVS than females, and age was not significantly associated with PVS counts.	WM-PVS volume Females: 241.2 (134) mm^3^ Males: 334.8 (192.4) mm^3^
Ballerini et al., 2020a	Older adults (*n* = 300, mean age = 72.7 years).	1.5T, T1, T2, T2*, FLAIR. Optimized frangi filtering.	PVS cluster size and width were associated with hypertension, stroke and WMH volumes. No associations were found between PVS measures and diabetes, hypercholesterolemia, or cardiovascular disease history.	CSO-PVS volume: 3410 mm^3^ PVS-VF of ICV: 0.22%
Ballerini et al., 2020b	Older adults (*n* = 381, mean age = 72.6 years).	1.5T, T2. Optimized frangi filtering.	PVS volumes and counts were associated with retinal microvascular changes.	WM-PVS volume: 3197 (1404.06) mm^3^
Valdés Hernández et al., 2020	Older adults (*n* = 300, mean age = 72.7 years).	1.5T, T1, T2, T2*, FLAIR. Optimized frangi filtering.	PVS volumes were indirectly associated with cognition.	CSO-PVS volume: 3290 (1460) mm^3^
Barisano et al., 2021b	Healthy adults (*n* = 897, mean age = 28.8 years).	3T, T1 divided by T2. NLMF and frangi filtering (EPC).	Age, body mass index and systolic blood pressure were associated with increased PVS ratios. PVS volumes were significantly greater when measured later in the day.	WM-PVS volume: 5,029 (2,153) mm^3^ PVS-VF of ICV: 1.14 (0.43) %
Sepehrband et al., 2021	Healthy adults (*n* = 423, mean age = 73.0 years); MCI patients (*n* = 173, mean age = 75.6 years).	3T, T1, FLAIR. NLMF and frangi filtering.	PVS-VF in the anterosuperior medial temporal lobe (asMTL) was smaller in MCI patients compared to control. asMTL-PVS VF shrinkage was associated with entorhinal neurofibrillary tau tangle deposition.	CSO-PVS VF^†^ Controls: ∼1.0% MCI: ∼1.2%
Ramirez et al., 2021	CVD patients (*n* = 152, mean age = 69.2 years).	3T, T1, T2, FLAIR. Modified Lesion explorer.	In CVD patients, PVS volumes were associated with increased sleep duration and time in bed.	Mean PVS volumes Whole brain: 43 mm^3^ WM: 22.5 mm^3^ BG: 16 mm^3^
Shen et al., 2021	PD patients (*n* = 40, mean age = 52.5 years); controls (*n* = 41, mean age = 49.8 years).	7T, T2. Manually segmentation.	PVS volumes in the midbrain and BG were associated with early Parkinson’s and PD markers.	Mean PVS volumes Left BG: 36.9 mm^3^ Right BG: 41.5 mm^3^ Left midbrain: 9.1 mm^3^ Right midbrain:13.5 mm^3^
George et al., 2021	MS patients (*n* = 3, age range = 32–35 years).	7T, T1, T2-TSE, SWI. Semi-automated frangi filtering.	MRI-visible PVS were predominantly periarterial spaces. Compared to controls, MS patients had a significantly greater proportion of perivenular spaces.	WM-PVS voxels: MS = 4,976 (282.6) Controls = 8,487 (2645.1)
Piantino et al., 2021	Veterans with mTBI (*n* = 56, median age = 32 years).	3T, T1, FLAIR. MAPS.	PVS counts and WM-PVS VF were significantly correlated with the number of mild traumatic brain injuries sustained.	WM-PVS volume: 351 (272.8) mm^3^ WM-PVS volume fraction: 1.3 (1) mm^3^/cm^3^
Donahue et al., 2021	PD patients (*n* = 285) and controls (*n* = 185, mean age = 59.8 years).	3T, T1. NLMF and frangi filtering	Global PVS VF was significantly larger in PD patients compared to controls and asymptomatic individuals. PVS VF in the banks of the superior temporal and the medial orbitofrontal regions were also significantly larger in PD subjects.	Global PVS VF PD: 0.74 (0.04) % Non-PD: 0.61 (0.04) %
Langan et al., 2022	COVID-19 patients (*n* = 10, mean age = 53.6 years); controls (*n* = 9, mean age = 51.2 years).	7T, T1, T2-TSE. PVSSAS.	PVS counts, but not volumes, were significantly higher in COVID-19 patients compared to controls. PVSSAS segmentation resulted in an inter-rater Dice score = 99.14%.	WM-PVS counts Controls: 3,232 (351) COVID19: 3,928 (866)
Ranti et al., 2022	MDD patients (*n* = 21, mean age = 35.0 years); healthy controls (*n* = 27, mean age = 39.7 years).	7T, T1, T2-TSE. PVSSAS.	In MDD patients, the number of traumatic events correlated with total PVS volumes. PVSSAS segmentation resulted in an inter-rater Dice score = 99.14%.	WM-PVS volume MDD: 7,517.99 mm^3^ Controls: 7,627.83 mm^3^
Lynch et al., 2022, Preprint	Healthy subjects (*n* = 1394, age range = 8–89.8 years)	3T, T1 divided by T2. NLMF and frangi filtering (EPC).	PVS measures were characterized across the lifespan. Measurements included VF, counts, solidity, and tortuosity.	WM-PVS VF range^†^: 0.5–2%
Barisano et al., 2022b	NASA astronauts (*n* = 24, mean age = 48.6 years); ROS cosmonauts (*n* = 13, mean age = 47.4 years).	3T, T1. NLMF and frangi Filtering.	WM-PVS volumes were significantly greater in astronauts who developed SANS, compared to those that didn’t.	WM-PVS volume postflight SANS: 1866.4 (769.7) mm^3^ Non-SANS: 1219.3 (567.5) mm^3^
Hupfeld et al., 2022	Astronauts (*n* = 15, mean age = 47.5 years); controls (*n* = 11, mean age = 42.3 years).	3T, T1. MAPS.	PVS counts were not associated with SANS. Novice astronauts exhibited significant changes in total PVS volumes after spaceflight, whereas experienced astronauts did not.	WM-PVS VF: 0.053 (0.044) %
Ramirez et al., 2022	PD patients (*n* = 133, mean age = 67.7 years)	3T, T1, T2-FLAIR, PD. Modified Lesion explorer.	Patients with smaller BG-PVS volumes were associated with MDS-UPDRS Parts I and II. Patients with larger BG-PVS volumes were associated with MDS-UPDRS Parts III and IV.	Median BG-PVS Volumes: 28.0–55.5 mm^3^ Counts: 6–9
Donahue et al., 2022	PD patients (*n* = 17, mean age = 64.5 years)	3T, T1. NLMF and frangi filtering	Larger PVS VF in the frontal lobe were associated with higher levels of choline-containing compounds. PVS VF in the anterior middle cingulate cortex was negatively correlated with concentrations of N-acetyl-compounds.	N/A
Kamagata et al., 2022	AD (*n* = 36), MCI (*n* = 44) and controls (*n* = 31). Mean age = 73 years.	3T, T1. NLMF and frangi filtering.	PVS-VF in both the WM and BG were significantly larger in AD patients compared to controls. MCI patients had significantly larger WM-PVS VF than controls.	WM-PVS VF Controls: 0.30 (0.02) % MCI: 0.29 (0.02) % AD: 0.28 (0.02) %
Barnes et al., 2022	Subjects with WMHs (*n* = 29, mean age = 71.9 years)	1.5T, T2, T1, FLAIR. Optimized frangi filtering and manual correction.	Topological relationships between WMH progression and PVS proximity were not statistically significant.	CSO-PVS volume Median: 7574 mm^3^ Range: 2,030–14,575 mm^3^
Jokinen et al., 2020	Subjects with WMHs (*n* = 639, age range = 65–84 years)	1.5T, T1. uResNet.	PVS measures were predictive of changes in processing speed, executive functions, memory, and VADAS score in the 3-year follow-up.	# PVS observed (% of cohort): 0: 5.0% 1–5: 46.1% 6–10: 22.5% ≥ 11: 26.4%
Zong et al., 2020	Healthy subjects (*n* = 45, age range = 21–55 years)	7T, T2, TSE. M^2^EDN.	BG-PVS counts, and VF were associated with age. Carbogen (a vasodilator gas) induced increased PVS-VF in the BG and WM.	PVS diameter: 0.69–0.71 mm VF: 0.05–0.33% Counts: 5.9–417
Wang et al., 2021	Middle-later aged subjects (*n* = 161, mean age = 60.4 years).	3T, T1, T2, SWI, T2-FLAIR. M^2^EDN.	Both quantitative and morphological measures of BG-PVS were associated with WMH volume and lacunes. Both WM and BG-PVS counts were associated with hypertension. Reported DSC for segmentation performance was 70%.	PVS volume, median (IQR) WM: 2370.6 (1520.6–3279.6) mm^3^ BG: 166.4 (80.1–412.4) mm^3^ PVS counts, median (IQR) WM: 490 (375–602) BG: 65 (39.5–155)
Huang et al., 2021	Healthy elderly subjects (*n* = 103, mean age = 59.5 years).	3T, T1, T2, T2-FLAIR, TOF-MRA. M^2^EDN.	Hypertension was associated with WM-PVS volume. Intracranial artery diameter was negatively correlated with WM-PVS volumes. Reported DSC for segmentation performance was 70%.	PVS volume, median (IQR) 2.6 (1.7–3.9) mL
Spijkerman et al., 2022	Cognitively impaired patients (*n* = 50, mean age = 62.9 years).	7T, T2-TSE. Sato filter and K-nearest neighbor algorithm.	PVS measures were extracted and mapped onto MNI152 space, generating heatmaps for PVS density, length, and tortuosity.	N/A

Almost all publications listed here have applied computational methods to facilitate the voxel-wise delineation of PVS in MRI. PVS measures reported by each publication are listed in the last column. It is clear that PVS measurements are highly heterogenous between studies that have applied different algorithms. The reported values are the mean (SD), unless otherwise specified. Listed first are the methods that utlize classical image filtering technique. In the second section are methods that have applied machine learning techniques. ^†^Derived visually from published graphs. AD, Alzheimer’s disease; MCI, mild cognitive impairment; VADAS, vascular dementia assessment scale; CVD, cardiovascular disease; PD, Parkinson’s disease; MDS-UPDRS, movement disorder society-unified Parkinson’s disease rating scale; MS, multiple sclerosis; mTBI, mild traumatic brain injury; MDD, major depressive disorder; NASA, national aeronautics and space administration; VF, volume fraction; CSO, centrum semiovale; asMTL, anterior superior medial temporal lobe; WMH, white matter hyperintensities; FLAIR, fluid attenuated inversion recovery sequence; TSE, turbo-spin-echo; TOF-MRA, time-of-flight magnetic resonance angiography; DSC, Sørensen-Dice similarity coefficient; NLMF, non-local means filtering; EPC, enhanced perivascular contrast; PVSSAS, perivascular Space semi-automatic segmentation tool.

In adolescence (12–21 years old), PVS appear to be bilaterally symmetric, and tend to visible more often in the frontal and parietal lobes compared to the temporal and occipital lobes (Piantino et al., 2020). Male adolescents (mean PVS count = 98.4) also had significantly greater WM-PVS counts than females (mean PVS count = 70.7) (Piantino et al., 2020). Thus, a spatial distribution of PVS enlargement, and sex differences arise early on. The biological mechanisms behind these observations, and whether this spatial distribution is constant or changes with age is unclear.

In young adults (21–37 years old), the mean diameter of PVS in the frontal, parietal-occipital, temporal lobes and subcortical nuclei (basal ganglia and thalamus) were significantly different, with those in the subcortical nuclei, wider than the three WM regions (Zong et al., 2016). Of these four regions, the parietal-occipital lobe exhibited the highest PVS volume fraction, and the temporal lobe, exhibited the lowest volume fractions. Moreover, heatmaps of the spatial distribution of PVS location, length, and tortuosity have been generated (*n* = 50, age range = 27–78 years old) (Spijkerman et al., 2022). Table 3 summarizes the publications that have explored the quantitative and morphological attributes of PVS.

**TABLE 3:** Summary of the PVS metrics that have been investigated by previous publications.

Study	Quantitative metrics			Morphological metrics			
		
	Volume	VF	Counts	Length	Width	Linearity	Tortuosity
Ramirez et al., 2015	✓						
Berezuk et al., 2015	✓						
Cai et al., 2015		✓					
Zong et al., 2016	✓	✓	✓	✓	✓		
Wang et al., 2016	✓		✓				
Niazi et al., 2018	✓	✓					
Piantino et al., 2020	✓	✓	✓	✓	✓		
Ballerini et al., 2020a	✓		✓	✓	✓		
Ballerini et al., 2020b	✓		✓	✓	✓		
Valdés Hernández et al., 2020	✓	✓	✓				
Zong et al., 2020		✓	✓		✓		
Barisano et al., 2021b	✓	✓					
Sepehrband et al., 2021	✓	✓					
Ramirez et al., 2021	✓						
Shen et al., 2021	✓		✓				
George et al., 2021	✓	✓					
Piantino et al., 2021	✓	✓	✓	✓	✓		
Donahue et al., 2021		✓					
Langan et al., 2022	✓	✓	✓	✓	✓		
Ranti et al., 2022	✓	✓	✓	✓	✓		
Lynch et al., 2022, Preprint		✓	✓		✓		✓
Barisano et al., 2022b	✓						
Hupfeld et al., 2022		✓	✓				
Barnes et al., 2022	✓						
Kamagata et al., 2022		✓					
Donahue et al., 2022		✓					
Ramirez et al., 2022	✓		✓				
Jokinen et al., 2020	✓						
Zong et al., 2020		✓	✓		✓		
Wang et al., 2021	✓		✓	✓	✓	✓	
Huang et al., 2021	✓	✓					
Spijkerman et al., 2022			✓	✓			✓

PVS measures can be broadly categorized as quantitative metrics (volume, count, and volume fractions), and morphological metrics (length, width, solidity, and tortuosity). Listed first are the methods that utlize classical image filtering technique. In the second section are methods that have applied machine learning techniques.

In a cohort of healthy older adults (*n* = 160, mean age = 60.4 years old), both WM and BG-PVS have been associated with cerebral small vessel disease markers, such as WMHs (Wang et al., 2021). BG-PVS volumes, counts, and width, and WM-PVS counts were associated with hypertension (Wang et al., 2021). Moreover, greater WM-PVS sizes were associated with presence of diabetes. In this cohort, the median number of PVS clusters of 490 and 65, consisting of median PVS volumes of 2,371 and 166 mm^3^, was detected in the white matter and basal ganglia, respectively (Wang et al., 2021).

Frangi-detected WM-PVS in the Lothian Birth Cohort (LBC1936, *n* = 533, mean age = 72.6 years old), found that mean PVS size (volume per cluster), length and width, as opposed to counts, to be associated with WMH (Ballerini et al., 2020a). PVS size and widths were also associated with hypertension and risk of stroke (Ballerini et al., 2020a). Notably, no relationship between PVS measures with diabetes, hypercholesterolemia or presence of cardiovascular disease was found (Ballerini et al., 2020a). Moreover, PVS volume, widths, and sizes were negatively correlated with retinal markers of microvascular health, such as the fractal dimension (a measure of vascular branching) and vessel diameter of arterioles and venules in the left eye (Ballerini et al., 2020b). Consistent with this result, WM-PVS volumes were found to be negatively correlated with intracranial artery diameter in a healthy cohort of old adults (*n* = 103, mean age = 59.5 years old) (Huang et al., 2021).

In the same cohort of community-dwelling individuals (LBC1936), the spatial proximity of WMHs with PVS was evaluated longitudinally (*n* = 29, mean age = 71.9 years old), with images collected over three sessions at 3-year intervals (Barnes et al., 2022). WMHs appeared to be adjacent to or co-localized with PVS, however, statistical analyses revealed no significant associations (Barnes et al., 2022).

A cross-sectional examination of the normative PVS traits across the human lifespan, has been conducted in the Human Connectome Lifespan Project (Lynch et al., 2022, Preprint). PVS were detected via EPC and Frangi filtering. PVS volume fraction, counts, and morphological measures such as diameter and solidity were found to be highly variable across all ages (Lynch et al., 2022, Preprint). Greater PVS burden in childhood was predictive of PVS expansion across the lifespan, especially in the temporal lobes, but was inversely correlated with growth in the limbic regions (Lynch et al., 2022, Preprint). WM-PVS volume fractions appeared to increase linearly across all ages, whereas PVS counts increased linearly until approximately the age of 50 years, whereby the number of detected PVS clusters decreased (Lynch et al., 2022, Preprint).

The findings are major contributions that provide reference ranges for comparison to pathological PVS changes. However, given that EPC vastly improves the detection of PVS in MRI, these ranges need to be adjusted to reflect the lower detection rate of image qualities typical in research or clinic (Sepehrband et al., 2019). Thus, the reported ranges are currently only applicable to similar datasets where the EPC pipeline has been used.

The study of perivascular spaces can be confounded by a number of variables. For example, age, hypertension and BMI are correlated with PVS measures (Francis et al., 2019; Barisano et al., 2021b). PVS volumes are also strongly related with intracranial volumes (ICV) (Ramirez et al., 2021). Generally, this is accounted for by modeling the PVS volume as a fraction of ICV or white matter volume (WM-PVS VF) (Piantino et al., 2020; Valdés Hernández et al., 2020; Sepehrband et al., 2021).

To complicate the issue, PVS volume has been shown to change throughout the day, being smallest in the morning and increasing throughout the day in a young and healthy cohort (*n* = 897, mean age = 28.8 years) from the Human Connectome Project (Barisano et al., 2021b). This suggests PVS enlargement is a physiological mechanism that is responsive to the needs of the neuronal environment throughout the day. This finding confounds the notion that perivascular spaces are enlarged in response to pathology (Kwee and Kwee, 2007). This leads to a complication for the imaging of PVS: to what extent are we observing physiological PVS enlargement in healthy glymphatic functioning shown in MRI, as opposed to PVS enlargement as a result of pathological damage-causing glymphatic dysfunction?

The complex relationship of MRI-visible PVS, with sleep also remains to be elucidated. Enlarged basal ganglia PVS has been associated with self-reported measures of total time spent in bed and time asleep (Ramirez et al., 2021). Poorer sleep efficiency and reduced duration of N3 sleep phase, measured by polysomnography, has also been correlated with enlargement of the BG-PVS (Berezuk et al., 2015). Given that the glymphatic system is most active during sleep, and PVS volumes demonstrate a diurnal or circadian rhythm, it is possible that brain-wide PVS measures are impacted not only by the time of MRI scan, but also by the duration and quality of sleep from the previous night (Xie et al., 2013).

In disease, abnormal PVS measures have been observed in patients with depression, COVID-19, traumatic brain injury, Multiple Sclerosis (MS), Parkinson’s disease (PD), mild cognitive impairment (MCI), and Alzheimer’s disease (AD) (Table 2; Cai et al., 2015; Ramirez et al., 2015, 2016, 2022; Niazi et al., 2018; Donahue et al., 2021, 2022; George et al., 2021; Piantino et al., 2021; Sepehrband et al., 2021; Shen et al., 2021; Kamagata et al., 2022; Langan et al., 2022; Ranti et al., 2022).

Significant higher PVS counts, but not volumes, have been found in COVID-19 patients, compared to controls (Langan et al., 2022). In patients with major depressive disorder, the severity of PVS volume enlargement has been associated with the number of traumatic events experienced in patients diagnosed with major depressive disorder (MDD) (Ranti et al., 2022). Notably, there were no significant differences in PVS measures (counts, density, and volumes) between MDD patients and controls (Ranti et al., 2022). Similarly, in U.S. military veterans, the number of mild traumatic brain injuries (mTBI) sustained was positively correlated with both PVS volumes and counts (Piantino et al., 2021). In this cohort of veterans, an interaction between mTBI events and sleep quality was significantly associated with PVS volumes (Piantino et al., 2021).

With the use of susceptibility weighted imaging (SWI) sequences, periarteriolar spaces can be distinguished from perivenular spaces (Bouvy et al., 2014). The majority of MRI-visible perivascular spaces tend to be associated with perforating arteries, as opposed to veins (Bouvy et al., 2014; George et al., 2021). In healthy controls (*n* = 3), only 10.3% of MRI-visible PVS clusters were found to be perivenular, with multiple sclerosis patients (*n* = 3) demonstrating a significantly greater proportion of visible perivenular space (15.19%) (George et al., 2021). Although the sample sizes were quite small, the results are consistent with the notion that enlargement of perivenular spaces may be associated with inflammation and inflammatory markers.

In PD patients, magnetic resonance spectroscopy has been used to investigate the regional concentrations of inflammatory neurometabolites with respect to PVS enlargement in different regions (Donahue et al., 2022). In the frontal white matter, higher PVS volume fractions were associated with higher levels of choline-containing compounds, an inflammatory marker (Donahue et al., 2022). Whereas, PVS-VF in the anterior middle cingulate cortex was negatively correlated with levels of N-acetyl containing compounds (Donahue et al., 2022). Additionally, PVS enlargement in both the midbrain and BG has been associated with motor symptom severity (Shen et al., 2021; Ramirez et al., 2022).

Furthermore, patients diagnosed with mild cognitive impairment (MCI) appear to have a higher WM-PVS volume fraction compared to controls (Niazi et al., 2018; Kamagata et al., 2022). However, no associations between PVS-VF, in either the WM or BG, were observed with hippocampal volumes, CSF levels of Aβ42, or neuropsychological scores (Kamagata et al., 2022). In contrast, reduced WM-PVS volume fraction in the antero-superior medial temporal lobe has been observed in MCI patients compared to controls (Sepehrband et al., 2021). Additionally, this shrinkage was associated with entorhinal neurofibrillary tau tangle deposition measured by positron emission tomography (Sepehrband et al., 2021).

In AD, both greater WM-PVS volume fractions and greater BG-PVS volumes have been observed in patients compared to age-matched controls (Cai et al., 2015; Ramirez et al., 2015; Kamagata et al., 2022). The enlargement of PVS has been widely implicated in a number of neurodegenerative diseases, exclusively in cross-sectional studies. Future longitudinal studies should examine the changes that occur in PVS with disease progression, especially in the later stages of disease. Such a study would be useful to examine the effect that disease has on the growth trajectory of PVS. For example, it is unknown whether PVS enlargement continues to occur in the later stages of brain and white matter atrophy, especially when neurodegeneration is accelerated by disease.

### Recommendations for future research and clinical applications

(1) Examine specific patterns of PVS enlargement, e.g., combinations of regionality, quantity (counts and volumes or volume fractions) and morphology (length, width, solidity).
(2) Focus on longitudinal studies especially given high inter- and intra-individual variability.
(3) Publish PVS counts, volumes, volume fractions, and time of MRI scans, field strength, and voxel size as descriptive variables, where possible.
(4) Publish SNR and CNR values, after applying preprocessing steps and immediately before PVS detection.

The individual labeling of PVS voxels enables more sophisticated research to be conducted in comparison to rating scales. Not only are we able to evaluate the severity of enlargement, but also analyze specific regions and morphology. This is important as the general finding of widespread PVS enlargement in response to various diseases does not offer a useful and highly specific biomarker of disease. Therefore, future research should examine specific patterns of PVS enlargement and shrinkage in response to disease severity. For example, shrinkage of PVS fraction in anterior-superior medial-temporal lobe (asMTL) of MCI patients (Sepehrband et al., 2021). It would be interesting to see whether the shrinkage of PVS volume fraction in the asMTL of MCI patients, alongside widespread PVS-VF enlargement in the WM is specific to MCI pathology and no other diseases including AD (Sepehrband et al., 2021). Although it is a paradoxical finding, it does provide support for more sophisticated analyses of PVS involving combinations of regionality and morphology. As indiscriminate and widespread enlargement of PVS in response to any type of pathology would unlikely be clinically useful.

Moreover, longitudinal studies will be most useful in assessing PVS changes specific to diseased states, given the very high inter-individual variability of PVS enlargement. There also appears to exist intra-individual variability, wherein PVS enlargement occurs in a diurnal manner, being smallest after sleep and gradually expanding throughout the day (Barisano et al., 2021b). The expansion between morning and afternoon is around 0.7 cm^3^ or 700 mm^3^. Given that the average adult brain contains around 5,000 mm^3^ of detectable PVS volume, diurnal variations may indeed play a significant factor to account for (±10% variation in PVS volume). Assuming an average of 8 h of sleep, this would imply that PVS volumes regularly expand on average 43.75 mm^3^ per waking hour (Barisano et al., 2021b). Therefore, studies should mention whether MRI scans were conducted during the morning or afternoon. This finding is especially important for PVS detection techniques of lower sensitivity, as fewer PVS are detected, findings of statistical significance may be a result of intraday variability (Ramirez et al., 2021). 009 To improve comparability, we recommend publishing average volumes and counts alongside volume fractions and MRI parameters and the resulting SNR and CNR. For a discussion of the MRI parameters that affect PVS visibility, please refer to Zong et al. (2016) and Barisano et al. (2022a).

Here we present one definition of SNR and CNR (Equation 2). Whilst there are many methods to derive them, the exact definition is unimportant. What is important is that the same definition is consistent across studies, and therefore useful as reference values. An SNR or CNR value calculated differently to the industry standard, should be explicit in its definition, as it does not serve as a useful reference, but instead potentially misleads readers by appearing to have a better or worse image quality than is present.

Currently there is no standard for PVS segmentation. This means different groups will likely end up with different CNR_*PVS*_ values due to the heterogenous PVS detection protocol. In addition to inter-group differences, there are large inter-rater differences. This will lead to substantial variation in reported CNR scores if PVS signal is used. Thus, the intensity of gray matter is used instead of PVS (Equation 2). The gray matter can be reliably segmented via established methods e.g., Freesurfer, FSL, and ANTs (Dale et al., 1999; Zhang et al., 2001; Avants et al., 2014). Another reason is that the PVS intensity appears to most closely resemble GM, as opposed to CSF intensity (Zong et al., 2020; Bernal et al., 2022). Thus, CNR measures based on GM intensity should be simple to compute and highly comparable between studies.

The application of high field (7T) MRI to elucidate the anatomic and pathological extent of PVS enlargement exhibits significantly better image quality than lower field strength scanners (1.5T and 3T) (Barisano et al., 2021a). In a study examining the differences in image quality between MRI scanners different field strengths, as expected, higher Tesla scanners outperformed 1.5T images in both SNR and CNR measurements (Magnotta and Friedman, 2006). However, the quality of the 1.5T images were the most stable between scanners, exhibiting a tighter range of SNR values, compared to the 3T and 4T images (Magnotta and Friedman, 2006). In some cases, the 3T images appeared to be no different to the 1.5T in terms of SNR. To employ a useful analogy, when measuring one’s weight on bathroom scales assembled by different companies, they may report different measurements. This is analogous to the problem of variability between MRI scanners or inter-scanner variability. Here, SNR can be used to measure the comparability between scanners.

Furthermore, when the typical bathroom scale is substituted with a highly sensitive industrial grade weighing scale, slight changes in weight and even involuntary movements are detectable. Consequently, when observing one’s weight on this sensitive instrument, the measurement might not settle on a single value, and instead display a constantly varying number. Similarly, PVS measurements derived from 7T scanners, may suffer from the same intra-scanner variability, although this remains hypothetical. In this case, CNR is useful as a metric for scanner performance.

To address the problem of variability between scanners, harmonization methods such as ComBat and NeuroHarmonize have been developed (Fortin et al., 2018; Garcia-Dias et al., 2020; Pomponio et al., 2020). A word of caution with these methods: by calculating the pooled average of image quality metrics and biasing all dependent variables in a consistent manner, the PVS metrics are effectively “harmonized.” However, this can lead to erosion of PVS measurements derived from images that have greater quality than the pooled average. Magnotta and Friedman (2006) suggest three potential solutions:

(1) Adjustment of scan parameters to provide similar SNR and CNR.
(2) Development of image processing methods that are unaffected by SNR and CNR differences.
(3) Statistical approaches to control for image quality, such as the use of SNR as a covariate in data analyses.

Taking the last idea one step further, Shannon’s entropy focus criterion (EFC), an image quality metric that measures the degree to which an image has been affected by motion artifacts such as ringing or ghosting, can be adjusted for in statistical analyses (Atkinson et al., 1997). EFC is easily derived with the MRI Quality Control (MRIQC) tool, and may be especially useful for PVS studies in disease cohorts with patients who struggle to remain motionless during MRI scans (Esteban et al., 2017).

## A case study of perivascular spaces in spaceflight associated neuro-ocular syndrome: How different algorithms can lead to vastly different results

Recently, two studies have been published examining the changes in perivascular spaces associated with being in a microgravity environment for 6 months. Both studies used 3T T1-weighted MRI to assess PVS changes in the brain after 6 months in space. Barisano et al. (2022b) examined participants within 2 weeks before and after 6 months of spaceflight. Hupfeld et al. (2022) conducted six scans, two before and four after. Both studies had age matched controls (Hupfeld et al., 2022). Barisano et al. (2022b) also had a group of astronauts with 2-week missions (*n* = 7). To detect and subsequently assess PVS quantities and morphology, Hupfeld et al. (2022) (*n* = 14) used MAPS in the white matter, whereas Barisano et al. (2022b) (*n* = 37+) applied non-local means filtering followed by Frangi filtering, in both the white matter and basal ganglia.

In each study, various non-overlapping hypotheses were tested. We will focus on the common hypotheses that were tested and as they relate to PVS:

(1) Whether previous experience in spaceflight was associated with different WM-PVS volumes or volumes fractions in pre-flight scans.
(2) Whether changes in WM-PVS volume or volume fraction, from pre- to post-flight were associated with previous days spent in space.
(3) Whether WM-PVS changes correlated with mission duration.
(4) Whether WM-PVS differences at baseline or pre-flight were associated with occurrence of spaceflight-associated neuro-ocular syndrome (SANS).
(5) Whether WM-PVS changes from pre- to post-flight were associated with occurrence of SANS.

In line with Hupfeld et al.’s (2022) suggestion of holdover effects due to previous spaceflight experience, Barisano et al. (2022b) reported high WM-PVS volumes in ROS cosmonauts who spent more time in space compared to NASA ISS astronauts (mean previous number of days in space = 266.3 vs. 88.8, mean preflight volume = 1976.4 vs. 1152.3 mm^3^, respectively), although this hypothesis was not directly tested. Hupfeld et al. (2022) found strong effect sizes for previous days spent in space with pre-flight PVS metrics including volumes, however, none of these reached significance (*r* = 0.60–0.71, *p* > 0.05) (Hupfeld et al., 2022).

Regarding the second hypothesis, Hupfeld et al. (2022) found that novice astronauts exhibited greater change in total PVS volume compared to experienced astronauts who exhibited a decrease in total PVS volume (*p* = 0.02). This association was not significant for other PVS metrics, but total PVS counts did show a trend toward significance (*p* = 0.068) (Hupfeld et al., 2022). In comparison, Barisano et al. (2022b) found that previous spaceflight experience was inversely correlated with changes in BG-PVS but not the WM-PVS. In other words, more time previously spent in space was associated with less change in PVS volumes of the BG due to further spaceflight exposure. Additionally, greater PVS volumes in both the BG and WM were found after 6 months of spaceflight (*n* > 37), which did not occur for astronauts who participated in 2-week missions (*n* = 7) (Barisano et al., 2022b). Conversely, Hupfeld et al. (2022) found no association of PVS volume fraction change with mission duration (*n* = 15, *p* > 0.05).

White matter-perivascular spaces volumes were larger pre- and -post flight for NASA astronauts who developed SANS, as opposed to those who didn’t, suggesting a link between PVS and SANS by Barisano et al. (2022b). Unlike Barisano et al. (2022b). and Hupfeld et al. (2022) found no difference in initial WM PVS volume fraction between SANS and non-SANS group (*p* = 0.41). However, neither study found the change in PVS volume before and after flight to be statistically significant different between the SANS and non-SANS group (Barisano et al., 2022b; Hupfeld et al., 2022).

In summary, of the five hypotheses that both studies examined, Barisano et al. (2022b) reached significance for three of the five, whereas Hupfeld et al. (2022) only one, with two tests trending towards significance. A number of factors could have impacted the failure to reach statistical significance such as random chance, high individual variability, small sample sizes, and methodological differences such as differing definitions for ventricular volumes. We suspect that a lower PVS detection sensitivity to be a major contributing factor to the failure to reach statistical significance.

The sensitivity of a PVS detection technique directly impacts the statistical power of the study. Lower detection rates will likely deprecate the ability of the study to rule out false negative hypotheses, i.e., have an increased Type 1 error, as fewer PVS are detected leading to lower PVS volumes. This seems to be the case in Hupfeld et al. (2022) wherein multiple tests did not reach statistical significance, but rather a trend was observed wherein *p*-values were close to the critical threshold.

**FIGURE 11:**
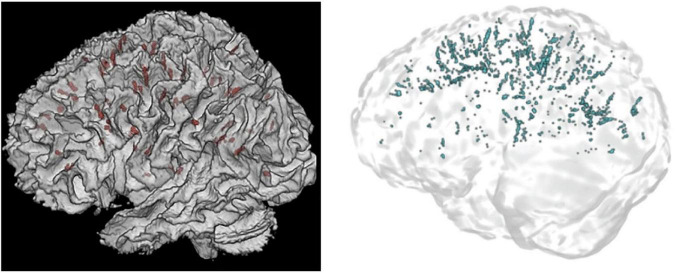
3D renders of PVS segmentations in astronauts from Hupfeld et al. (2022) **(left)** and Barisano et al. (2022b) **(right)**. On the left, PVS are displayed in red, whilst on the right PVS are blue. Reproduced under the Creative Commons Attribution 4.0 International License (https://creativecommons.org/licenses/by/4.0/).

In Figure 11, it is visually evident that the Frangi approach is much more sensitive than the approach taken by Barisano et al. (2022b) and Hupfeld et al. (2022). Furthermore, the differing PVS detection ability between the approaches can be elucidated by looking to the published PVS volumes. Whilst Barisano et al. (2022b) reported average PVS volumes (mm^3^), Hupfeld et al. (2022) reported PVS volumes as a fraction of white matter volume (mm^3^/cm^3^). The highest mean volume fraction reported by Hupfeld et al. (2022), was 0.53 mm^3^/cm^3^, occurring post-flight at scan six (return +180 days) in all astronauts. If a liberal white matter volume of 600 cm^3^ is assumed, this would imply that a mean of 318 mm^3^ of PVS volume was detected (Farokhian et al., 2017). In comparison, the lowest mean WM-PVS volume reported by Barisano et al. (2022b) is 768.8 mm^3^. This is more than double the volume reported by Hupfeld et al. (2022), despite the higher resolution and MRI coils utilized which presumably leads to better detection of PVS voxels. However, without SNR or CNR values published, it is unclear whether one study had better image quality than the other despite both conducting 3T, T1-weighted MRI scans.

These values can be further contextualized with published normative ranges of PVS volume fractions averaging 1.14%, or mean PVS volume of 5029.39 mm^3^ for healthy adults (*n* = 897, mean age = 28.8 years) from the Human Connectome Project, found using Frangi filtering, similar to Sepehrband et al. (2019; Barisano et al., 2021b; Lynch et al., 2022, Preprint). Despite being significantly younger than the cohort of studied astronauts, the average PVS volume was significantly greater (5029.39 mm^3^) in the former group compared to the PVS volumes of 318 mm^3^ and 786.8 mm^3^, published by Barisano et al. (2022b) and Hupfeld et al. (2022), respectively. Importantly, the normative PVS values were determined via the combination of co-registered T1 and T2 images (EPC) to substantially improve the CNR of PVS making these comparisons unfair (Sepehrband et al., 2019; Barisano et al., 2021b). It does, however, highlight that normative ranges for PVS distributions are yet to be established for commonly studied MRI sequences, in this case, for single-modality 3T, T1-weighted images, and that preprocessing techniques will have great impact on the subsequent result. Specifically, the EPC preprocessing pipeline of combining T1 and T2 images may have improved PVS visibility by a factor of six compared to unimodal detection (Sepehrband et al., 2019), again highlighting the importance of publishing image quality metrics after preprocessing steps and before PVS detection.

In comparison, Hupfeld et al. (2022) employed MAPS to segment perivascular spaces, an algorithm that has been involved in several publications. Hupfeld et al. (2022) states that the “group mean PVS values [of the astronauts] fall within the range of … a cohort of male veterans with a history of traumatic brain injury.” For this cohort of veterans (*n* = 56, mean age = 32 years), the mean PVS volume fraction of WM was 1.3 (*SD* = 1.0) mm^3^/cm^3^ (Piantino et al., 2021). Moreover, PVS characterization via mMAPS (multi-modal MAPS) in the white matter of healthy male adolescents (*n* = 56, mean age = 16 years), uncovered a mean total PVS volume of 334.8 (192.4) mm^3^ or mean PVS volume fraction of WM = 0.7 (0.4) mm^3^/cm^3^ (Piantino et al., 2020). Using mMAPS, Boespflug et al., found a mean PVS volume of 303.0 (267.7) mm^3^ in healthy old adults (*n* = 14, mean age = 85.3 years) (Boespflug et al., 2018). These findings are greater than the largest value reported for Hupfeld et al.’s (2022) cohort of astronauts (mean PVS volume fraction = 0.53 mm^3^/cm^3^, mean age = 47.46 years).

Whilst these values are roughly in line with each other, it is not consistent to find less PVS volumes in older cohorts, given that age is one of the most established predictors of PVS volume (Lynch et al., 2022, Preprint). It can be argued, however, that the results differ due to the multiple MRI modalities available in each case. Piantino et al. (2020) applied MAPS with T2, T1, and FLAIR modalities to detect PVS in adolescents. Whereas only T1-weighted images were available to Hupfeld et al. (2022). This could explain the larger PVS volumes detected in adolescents compared to significantly older but healthy astronauts.

## Conclusion

Taken together, quantitative measures of perivascular spaces that have been published are highly inconsistent, not only between studies using different detection methods (e.g., MAPS and Frangi filtering) but also within studies applying the same PVS segmentation technique. This can be due to a variety of reasons, such as preprocessing methods and heterogenous image qualities. Whilst we are critical of the results published, this in no way invalidates previously published results. They merely reflect the fact that different approaches to the same problem will likely lead to different outcomes, e.g., less sensitive algorithms will likely yield less significant outcomes. Nor does this review call for a complete overhaul of established methods. Huge amounts of time and effort are dedicated in order to develop a functional pipeline for automated PVS segmentation. The present review also highlights the poor comparability of results between studies due to differing PVS detection techniques. We therefore, make recommendations to help solidify the foundations for future PVS research as they relate to developments of new detection methods in MRI and subsequent applications. Development of novel segmentation methods should focus on the Sørensen Dice similarity coefficient as the main measure of model performance. In the application of these models, publication of image quality metrics alongside MRI scanner properties, and descriptive statistics of PVS measures should facilitate comparability between studies.

## Author contributions

WP wrote the manuscript. All authors contributed to edits, revisions, and approval for submission.
